# Association of Cerebral Small Vessel Disease With Gait and Balance Disorders

**DOI:** 10.3389/fnagi.2022.834496

**Published:** 2022-07-08

**Authors:** Chen Su, Xiaoyu Yang, Shuqi Wei, Renliang Zhao

**Affiliations:** Department of Neurology, The Affiliated Hospital of Qingdao University, Qingdao, China

**Keywords:** gait and balance disorders, cerebrovascular disease, white matter hyperintensity, lacunar infarction, cerebral microbleeds, enlarged perivascular space, brain atrophy

## Abstract

Cerebral small vessel disease (CSVD) is a common cerebrovascular disease and an important cause of gait and balance disorders. Gait and balance disorders can further lead to an increased risk of falls and a decreased quality of life. CSVD can damage gait and balance function by affecting cognitive function or directly disrupting motor pathways, and different CSVD imaging features have different characteristics of gait and balance impairment. In this article, the correlation between different imaging features of sporadic CSVD and gait and balance disorders has been reviewed as follows, which can provide beneficial help for standardized management of CSVD.

## Introduction

**Figure 1:**
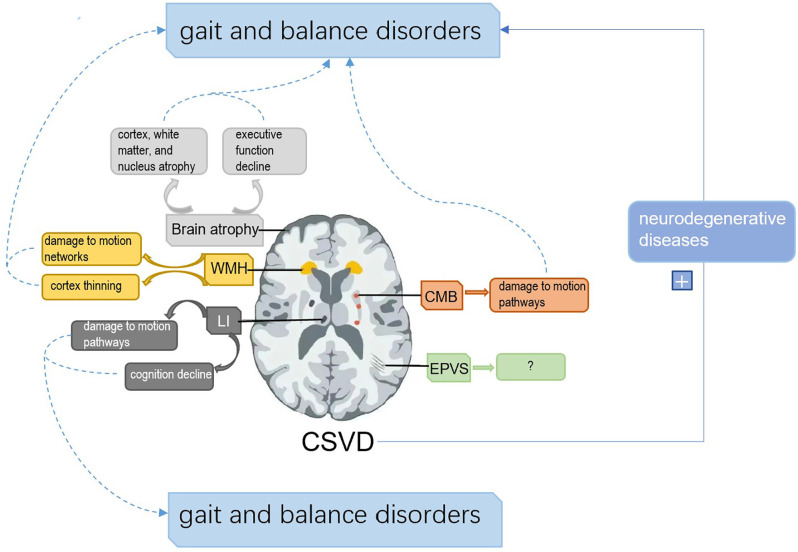
The framework of the associated mechanisms between cerebral small vascular disease (CSVD) and gait and balance disorders. **(1)** White matter hyperintensities (WMH) can disrupt gait and balance by interfering with motion pathways and cortical thickness. **(2)** Lacunar infarctions (LI) impair gait and balance by interfering with cognition and motion pathways. **(3)** Cerebral microbleeds (CMB) affect gait and balance by damaging motion pathways. **(4)** Brain atrophy can disrupt gait and balance by damage to cognition, nucleus atrophy related to motion control, and cortex and white matter atrophy. **(5)** Enlarged perivascular spaces (EPVS) have shown no direct evidence to disrupt gait and balance. **(6)** CSVD can aggravate the impairment of gait and balance by affecting neurodegenerative diseases.

Cerebral small vessel disease (CSVD) is a pathological process of cerebral arterioles, capillaries, and venules caused by a variety of factors. CSVD appears on magnetic resonance imaging (MRI) as white matter hyperintensities (WMH), cerebral microbleeds (CMB), lacunar infarctions (LI), enlarged perivascular spaces (EPVS), and brain atrophy (Pantoni, 2010; Wardlaw et al., 2013a). Sporadic CSVD is closely correlated with age, with prevalence increasing from approximately 5% in people aged 50 to nearly 100% in people aged 90 (de Leeuw et al., 2001). CSVD is a major cause of cognitive impairment, dementia, and stroke, which is a huge burden on public health (Debette and Markus, 2010). Gait and balance disorders are the second most common problem in CSVD after cognitive impairment (Okroglic et al., 2013). Gait disorders are common in older adults and can lead to falls and functional dependence, increasing the risk of hospitalization and death (van der Holst et al., 2016; Bower et al., 2019). A study conducted by Wei et al. (2017) showed that gait and balance function were independent predictors of the risk of falls in patients 1 month after stroke, suggesting that appropriate gait training may be one of the key factors in preventing falls in patients with stroke. We aim to assist the standardized management of CSVD by discussing the pathogenesis, diagnosis, and treatment of gait and balance disorders associated with sporadic CSVD. The mechanism of the association between sporadic CSVD and gait and balance disorders is summarized as follows (Figure 1).

## Correlation Between CSVD and Gait and Balance Disorders

Gait and balance are considered to be multisystem interactions, which suggests that clinical gait and balance problems may arise from different pathological mechanisms, such as attention, planning, visuospatial, and motor processes, and are closely related to executive function in the cognitive domain (Horak, 2006). The study of Cai et al. showed that gait disorders in elderly patients with CSVD are mediated by cognitive function impairment (Cai et al., 2021). Gait requires the supervision of cognitive function, especially executive function, and patients need the brain to allocate attention to control gait and balance. The decline in cognitive ability will damage their balance control ability (Yip et al., 2014; Cai et al., 2021). CSVD can cause extensive neural network damage by damaging important fiber bundles and loops, resulting in impaired visual, cognitive, sensory, and motor functions, especially in the frontal lobe, temporal lobe, basal ganglia, and other parts related to executive function, which will lead to a more obvious decline in executive function (Chao et al., 2009). Zhou et al. (2020) showed that gait and balance disorders in CSVD patients were related to the sensorimotor network and frontoparietal network as well as abnormalities in auxiliary motor areas. Other studies have shown that CSVD disrupts the cortex-striatum- globus pallidus-thalamic-cortical circuit, leading to depressive states, and poststroke depression also increases the risk of gait disorders (Wei et al., 2017; Rensma et al., 2018).

### Association of WMH With Gait and Balance

WMH are a type of sporadic small vascular disease, and axon loss and demyelination are the causes of WMH, which may be the result of chronic ischemia caused by CSVD (Staszewski et al., 2015). It is also believed that hypoperfusion is related to cerebral vascular autoregulation changes or blood–brain barrier dysfunction (Simpson et al., 2007), inflammation, and amyloid vascular disease (Gouw et al., 2011). 20% of WMH can be found in adults in their 60s and up to 94% in those in their 80s (Garde et al., 2000). On imaging, WMH show high signals on T2-weighted sequences and equal signals or low signals on T1-weighted sequences (Fujishima et al., 2014). WMH can be divided into periventricular white matter hyperintensities (PVWMH) and deep white matter hyperintensities (DWMH). Traditional imaging techniques or semiautomated techniques are used to measure the volume of white matter lesions. New methods, such as magnetic resonance diffusion tensor imaging (DTI) and voxel-based morphology analysis (VBM), can also be used to analyze the integrity of white matter. One study confirmed that WMH scores and total CSVD scores were associated with poorer gait speed and body balance in healthy older adults (Pinter et al., 2017). In patients with vascular cognitive impairment, gait and balance disorders were mainly characterized by reduced arm swing, stoop, reduced step length, and broad base gait, which were only associated with PVWMH volume and white matter integrity but not with other radiological markers of CSVD (Kim et al., 2016). The LADIS study confirmed that the severity of WMH in the deep frontal lobe and PVWMH significantly impaired balance function in the elderly population, which supported the hypothesis that the disruption of motor circuits in the subfrontal cortex caused balance disorders (Blahak et al., 2009). DTI provides an indicator of the internal microstructure integrity of neural networks and can reflect the loss of white matter integrity (Pasi et al., 2016). A study using DTI found that stride length decline was associated with white matter atrophy and an increase in mean white matter radial diffusivity and mean diffusivity and a decrease in mean fractional anisotropy (FA), with the strongest association found in the corpus callosum and radial crown fibers (van der Holst et al., 2017). The corpus callosum white matter connects the frontal, parietal, and occipital cortexes, and fibers from these areas reverse into the radiative crown, which contains projective fibers involved in motor pathways and thus plays a key role in motor function (Jang, 2009). In another study using DTI, total white matter lesions in CADASIL patients only affected stride length on a single task (Finsterwalder et al., 2019). PVWMH are more likely to cause cognitive impairment than DWMH and executive dysfunction due to the concentration of a large number of neurons and fibers related to learning, memory, and cognition, and the white matter bundle crossing periventricular white matter is denser (Onteddu et al., 2015). White matter injury, especially DWMH, also increased the likelihood of poststroke depression (Fujishima et al., 2014). However, no studies have directly shown that WMH affect gait independently by affecting cognition or depression. A study using the VBM technique showed that the bilateral frontal and periventricular white matter corresponding to the major anterior projection fibers (thalamic radiation, cortical motor tracts) and adjacent association fibers (corpus callosum, superior fronto-occipital fasciculus, short association fibers) showed the greatest correlation with poor gait (Srikanth et al., 2010). This suggested that white matter injury can lead to an age-related decline in gait and trunk balance stability by disconnecting the motor network composed of these fiber bundles. Another study using the VBM technique showed that fibers associated with gait and balance disorders crossed periventricular white matter more densely than deep white matter and that the cortical regions connected by these fibers overlapped with cortical thinning regions associated with gait performance. The mediation analysis showed that PVWMH mediated gait disorders through average FA or cortical thickness (Kim et al., 2016). We can determine that WMH affect gait and balance through the following mechanisms: (1) direct damage to motion-related neural networks; and (2) destruction of white matter integrity leading to thinning of the related cortex.

### Association of LI With Gait and Balance

LI, which account for 25% of all acute ischemic strokes, are radiographically characterized by round, oval, or tubular infarcts with a diameter <20 mm, hypersignal on diffusion weighted imaging (DWI), hypersignal on T2-weighted and fluid-attenuated inversion recovery (FLAIR), and hypersignal on T1. They usually occur in the basal ganglia, inner sac, thalamus, corona radialis, centrum semiovale, and brain stem (Wardlaw et al., 2013a; Li et al., 2018). LI are usually silent and found in 20%–50% of older adults (Vermeer et al., 2007). While many LI lack acute symptoms, their presence in large numbers has been associated with dementia, cognitive impairment, gait impairment, and an increased risk of stroke (Shi and Wardlaw, 2016). A study of healthy community populations showed that LI were associated with slower gait speed and balance disorders (Smith et al., 2015). LI located in the frontal lobe and thalamus were associated with lower gait speed, and stride length was more sensitive than stride speed as an outcome indicator (de Laat et al., 2010), which can be related to the involvement of the frontal-basal ganglia-thalamic-cortex circuit in gait motor function (Bazner et al., 2000).

LI are an important predictor of cognitive impairment in CSVD with significant spatial distribution characteristics, especially the anterior medial thalamus LI, which are associated with impaired information processing speed and may lead to cognitive impairment by disrupting connections to the prefrontal cortex (Benjamin et al., 2014, 2018). A study linked LI in the frontal lobe to motoric cognitive risk syndrome (MCR; Wang et al., 2016). MCR is a predementia syndrome characterized by cognitive complaints and slow gait in elderly people without dementia (Verghese et al., 2014). LI in the frontal lobe may cause MCR by disrupting neural networks based on the frontal lobe that assist memory and gait function (Takakusaki, 2013). Studies have shown that LI interact with WMH, and the presence of LI can aggravate the impairment of gait and postural stability in patients with WMH, which may be the result of a higher brain injury load leading to more easily impaired functions (Choi et al., 2012). In addition, deep white matter LI are also associated with the severity and fluctuation of depressive symptoms, which may be related to the disruption of the emotional circuit (Grool et al., 2013). However, whether LI can affect gait and balance by affecting WMH or depression is unclear.

In conclusion, LI can affect gait and balance function through the following two mechanisms: (1) direct damage to motor pathways, such as the frontal lobe and basal ganglia; and (2) MCR by influencing cognition.

### Association of CMB With Gait and Balance

CMB are circular or elliptical uniform low signal regions with a clear boundary and a diameter of 2–5 mm and a maximum of 10 mm on gradient recalled echo (GRE) and susceptibility weighted imaging (SWI). CMB have been shown to occur in 23.5% of healthy elderly populations (Pasi et al., 2017). CMB were found in 17.8% of patients aged 60–69 years and 38.3% of patients aged over 80 years, with the prevalence fluctuating by approximately 5% (Cordonnier et al., 2007; Ding et al., 2015). The CMB has the same spatial distribution characteristics as LI and is mostly located at the corticocortical subcortical junction and deep gray or white matter in the hemispheres, brainstem, and cerebellum (Wardlaw et al., 2013b). Currently, there are few studies on the association between CMB and gait and balance disorders. For instance, de Laat et al. (2011) demonstrated for the first time that CMB located in the basal ganglia, thalamus, and frontal lobe were associated with gait and balance disorders in elderly patients without dementia, independent of other coexisting CSVD markers, and these regions were associated with gait control. Gait and balance disorders were mainly represented by shorter stride length and decreased trunk balance (de Laat et al., 2011). This suggested that CMB can directly damage the site and thus disrupt motor pathways. However, most studies since then have shown no direct link between CMB and gait and balance. A cross-sectional study in Japan showed that short standing time on one leg was independently associated with the number of *lacunes* and CMB and was associated with decreased cognitive function (Tabara et al., 2015). The CMB is an important factor in the decline in cognitive function (Lei et al., 2013). The presence of CMB in the frontal and temporal lobes may be associated with decreased nonverbal memory, visual-spatial memory, and psychomotor speed (van Norden et al., 2011). The existence of the basal ganglia CMB may be associated with attention and computing ability, and the existence of the thalamus CMB may be associated with independent overall cognition (Yakushiji et al., 2008). Whether CMB can indirectly lead to gait and balance disorders by influencing cognition remains to be further studied. CMB interact with WMH, and similar to LI, the presence of CMB also increases the negative effects of WMH on gait and balance (Choi et al., 2012). However, whether CMB can affect gait and balance through WMH is unknown.

As discussed above, CMB can affect gait and balance through direct damage to motor pathways associated with gait and balance control.

### Association of Brain Atrophy With Gait and Balance

Brain atrophy is characterized by a reduced brain volume on CT or MRI and is unrelated to local volume reduction caused by trauma or infarction. Atrophy is characterized by enlarged ventricles and widened sulci gyrus (Muller et al., 2011; Wardlaw et al., 2013b). Many imaging studies have reported the presence and severity of CSVD associated with brain atrophy, including global, cortical, subcortical, central (enlarged ventricles and basal ganglia), midbrain, and hippocampal atrophy (Appelman et al., 2009, 2010; Aribisala et al., 2013). A study of CSVD and gait disorders showed that gait scores were associated with thinning of the bilateral motor area, premotor area, dorsolateral prefrontal area, anterior cingulate area, and lateral temporoparietal occipital cortex. Thinning of the mean cortex was associated with gait severity, stooped posture, magnetic gait, small step, shuffling, and broad based gait (Kim et al., 2016). Brain white matter atrophy is associated with decreased gait speed, step length, and step frequency in community residents aged 60–86 years, hippocampal atrophy is associated with decreased gait speed and step length, and total gray matter atrophy is associated with decreased gait rhythm in patients with cerebral infarction (Callisaya et al., 2013). Basal ganglia LI, caudate nucleus atrophy, and global brain atrophy are associated with bradykinesia and balance disorders in healthy subjects aged 45–84 years (Camarda et al., 2018). Brain atrophy predicts cognitive decline, hippocampal volume correlates with changes in executive function performance, and frontal and temporal gray matter predicts changes in the verbal episodic memory performance (Aljondi et al., 2019). A study using PET technology found that reduced glucose metabolism in the posterior cingulate cortex and primary sensorimotor cortex was associated with lower gait performance (reduced step speed, reduced stride frequency) and executive function, suggesting that gait control and executive function may share the same neural substrate (Sakurai et al., 2017). Mediation analysis confirmed that thalamic atrophy mediated the effect of CSVD on walking speed in elderly people, and CSVD affected walking performance by destroying thalamic integrity (Su et al., 2018).

In summary, brain atrophy can affect gait and balance through the following two mechanisms: (1) atrophy of the cortex, white matter, and nucleus; and (2) reduced executive function.

### Association of EPVS With Gait and Balance

Perivascular spaces (PVS) are formed when the arterioles and venules pass through the brain parenchyma from the subarachnoid space. PVS are important drainage channels for the interstitial fluid of the brain. When the PVS is enlarged, EPVS are seen as punctate or linear high intensity on T2-weighted MRI, and diameters are usually no more than 3 mm (Wardlaw et al., 2013b; Potter et al., 2015). The incidence of EPVS was 79.9%, and the incidence of EPVS in the basal ganglia was slightly higher than that in white matter (Zhang et al., 2014). EPVS are generally associated with other morphological features of CSVD, such as WMH and LI, but not with brain atrophy (Kwee and Kwee, 2007; Zhu et al., 2010). At present, there are few reports on EPVS related to gait and balance, and only a few studies have reported the relationship between EPVS and Parkinson’s disease and Alzheimer’s disease populations. Basal ganglia EPVS were related to the poor prognosis of Parkinson’s disease motor symptoms and the tremor score of Parkinson’s disease patients (Wan et al., 2019; Chung et al., 2021). Basal ganglia EPVS were also a biomarker of CSVD in patients with senile dementia and higher in patients with vascular dementia than in Alzheimer’s disease and healthy volunteers. Poor scores of nonverbal reasoning and global visuospatial tasks were significantly associated with EPVS load in healthy people (Hansen et al., 2015).

At present, the relationship between EPVS and gait and balance function is unclear and requires further study.

### Association of Total CSVD Burden With Gait and Balance

The above explores the relationship between individual imaging markers of CSVD and gait and balance. Total CSVD burden (0–4 points): ≥1 cavity is one point; DWMH score is 2 or 3 or PVWMH score is 3 points; moderate to severe EPVS (2–4 points) was scored as one point; CMB ≥1 score is 1 point (Wardlaw et al., 2013b; Staals et al., 2015). A growing body of research shows that summarizing the total CSVD load of individual markers of CDVD on the load scale may better reflect the overall effect of CSVD on the brain (Staals et al., 2015; Pinter et al., 2017). However, there have been few studies on the effect of the total burden of CSVD on gait and balance. A study of elderly subjects with vascular risk factors showed that concurrent gait function as measured according to the Parkinson’s disease assessment scale was associated with the overall CSVD burden. This is the first study to examine concurrent gait function and overall CSVD burden, although the CSVD burden score does not include EPVS (Hatate et al., 2016). A study of 678 elderly healthy subjects in the community found that the total WMH and CSVD scores were independently associated with slower gait speeds in the 6-meter walk test (Pinter et al., 2017). The results of this cross-sectional study need to be validated by large prospective studies. However, in a recent prospective study of total CSVD burden and minor stroke patients, CSVD scores at 3 years of follow-up were not associated with objective gait and balance disorders but only with subjective mobility disorders (Loos et al., 2018). The relationship between the overall burden of CSVD and gait and balance needs further study.

## CSVD and Neurodegenerative Diseases

There is growing evidence of an interaction between CSVD and neurodegenerative diseases, and we explore the effects of both on gait and balance below.

### Parkinson’s Disease

Parkinson’s disease (PD) is a neurodegenerative disorder that causes decreased motor capacity and nonmotor symptoms (Pfeiffer, 2016). PD and CSVD are both age-related diseases (De Virgilio et al., 2016), and a large number of relevant studies in recent years have continued to confirm the prevalence of concomitant CSVD in patients with PD. The study found that 76% of patients with PD had concomitant CSVD (Song et al., 2017). PD and CSVD have a common pathological inflammatory response, and studies have confirmed that CSVD can participate in the development of PD through an inflammatory response (Lenart et al., 2016; La Vitola et al., 2018). Bohnen et al. (2011) found that the severity of WMH was positively correlated with the axial motor symptom score and bradycardia score of patients with PD. Recent studies have also confirmed that the presence of WMH is an independent risk factor for postural gait disorders in patients with PD (Wan et al., 2019). Another study found that in patients with PD, the postural instability disorder type with WMH was more severe than the tremor type, which further confirmed that WMH were associated with axial dyskinesia of PD (Malek et al., 2016). It has been suggested that WMH, especially frontal white matter lesions, may disrupt the integrity of the frontal-striatal neural circuit and the frontal parietal circuit, thereby aggravating the damage to axial motor function in patients with PD (Lee et al., 2009). There are few studies on the correlation between LI and CMB and motor symptoms in PD patients. A cross-sectional study conducted by Zhang et al. (2016) showed that patients with PD who had asymptomatic LI in the striatum had severe disruption of substantia nigra structures. The study suggests that striatal LI in patients with PD may disrupt fibrous bundles in the striatum-nigra loop, which in turn affects the severity of motor symptoms in patients with PD. A cross-sectional study was grouped according to whether the subjects had CMB, and the results suggested that CMB may have no effect on motor symptoms in patients with PD (Kim et al., 2015).

### Alzheimer’s Disease

Alzheimer’s disease (AD) is a progressive neurodegenerative disease of the brain in which clinical symptoms gradually develop from cognitive impairment to dementia (Vinters, 2015). It is well known that adult patients with AD have balance and gait deficits compared to non-AD patients (Kato-Narita et al., 2011; Suttanon et al., 2012; Gras et al., 2015). As cognitive dysfunction progresses, physical function in patients with AD declines, and deficits in balance and gait become more pronounced. This is associated with impaired executive function in patients with AD (Scherder et al., 2007). Many studies have shown that CSVD increases the risk of developing AD, and pathological signs of AD can also be present in both vascular dementia and CSVD (Henry-Feugeas, 2008). Although the underlying mechanism of CSVD-induced AD pathology is unclear, it could be explained by myelin fibrosis degeneration and neuronal death due to chronic cerebral hypoperfusion or blood–brain barrier (BBB) disruption from CSVD (Pantoni, 2010; Kim H. W. et al., 2020). WMH has been widely accepted in imaging associations with aging and AD (Chen et al., 2011). For example, a longitudinal study showed that a high burden of WMH was associated with an increased risk of developing AD over a 5-year follow-up period (Ye et al., 2019). One cohort study showed that overall WMH load was associated with AD risk factors in subjects whose cognitive function was not impaired. The main drivers of this association are age and high blood pressure (Salvado et al., 2019). The role of CMB and LI in AD is unclear. A cross-sectional study found that CMB was associated with vascular burden and the diagnosis of AD (Caballero et al., 2020). A meta-analysis showed that CMB did not affect the course of AD (Liu et al., 2018). One cross-sectional study showed that elevated levels of LI were associated with increased levels of Aβ42 in vascular dementia and decreased levels of Tau in AD (Kester et al., 2014). Microinfarction is closely related to AD, and the number of microinfarcts is related to overall cognitive ability (van Rooden et al., 2014). Current studies do not support the role of EPVS in the early pathogenesis of AD (Gertje et al., 2021). Brain atrophy is the most important imaging morphological feature of AD (Guo et al., 2014), but the link between cerebral atrophy in the context of CSVD and AD remains unclear.

### Multiple Sclerosis

Multiple sclerosis (MS) is a chronic inflammatory demyelinating disorder of the central nervous system in which neurodegeneration leads to long-term disability (Lassmann et al., 2007). MS leads to gait and balance disturbances and falls (Chee et al., 2021). Age is a major determinant of MS progression, onset, and disability (Tutuncu et al., 2013). CSVD is a common age-related cerebrovascular disease (de Leeuw et al., 2001). An autopsy study showed severe small artery disease in the brain of MS patients, and MS patients had a high burden of CSVD (Geraldes et al., 2020). Multiple lines of evidence suggest that the interaction between MS and CSVD may affect MS-related neurodegeneration. The chronic inflammatory environment of MS can increase the vulnerability of the cerebral vasculature to the vascular risk factors, thereby exacerbating CSVD (Scalfari et al., 2014). At the same time, CSVD can cause oligodendrocyte damage, white matter damage due to demyelination, and neuronal loss through hypoperfusion and BBB dysfunction, and this “vasculo-neuronal inflammatory” model can be applied to MS (Wardlaw et al., 2013a; Geraldes et al., 2017). Tissue hypoxia is associated with neurological deficits (Davies et al., 2013), and the presence of tissue hypoxia associated with inflammation in MS (Desai et al., 2016). The presence of CSVD exacerbates the hypoxic environment, thereby exacerbating tissue damage (Martinez Sosa and Smith, 2017). However, a recent study of genes showed no association between WMH volume and genetic polymorphisms in MS. This study concluded that ischemic white matter damage and MS differ in the nature of their primary damage. Inflammation acts through different pathways and there are no common physiological mechanisms (Brown et al., 2019). However, the MS genetic data used in this study focused on disease risk rather than the degree of white matter lesions in the brain. The above suggests that CSVD can exacerbate MS. However, no trials have directly examined the relationship between CSVD and gait and balance deficits in MS patients.

## Gait and Balance Function Evaluation Method

The assessment of gait and balance function is crucial to evaluating the association between CSVD and gait and balance. The gait disorders caused by CSVD are different from the characteristics of Parkinson’s disease, so the traditional Parkinson’s disease motor assessment scale is unsuitable. The Short Physical Performance Battery (SPPB), Berg Balance Scale (BBS), Timed Up and Go Test (TUG), Tinetti Mobility Test (TMT), and other semiquantitative scales have been used more widely to measure gait and balance function in clinical studies. At present, the rise of some new measurement methods different from traditional scales, such as wearable electronic devices, can obtain more comprehensive and detailed gait parameters for more accurate evaluation.

### SPPB

The SPPB is a rapid, objective three-part physical function test that is a comprehensive indicator of walking speed, standing balance, and sitting performance. It has good predictive validity and broad clinical applicability. It includes a normal walking speed of more than 4 m, five sit-stand tests, and a balance test. The score of each task is four points, and the scores of the three tests are added to obtain a total, with the maximum value being 12 and the minimum value being 0. The higher the total score is, the higher the functional level is (Guralnik et al., 2000; Perera et al., 2006). The SPPB has been shown to be predictive of adverse outcomes, including all-cause mortality, disability, and hospitalization (Volpato et al., 2011; Minneci et al., 2015; Pavasini et al., 2016), and SPPB scores have been shown to be independently associated with falls among hospitalized patients and elderly community residents (Lauretani et al., 2019; Welch et al., 2021). The SPPB has been primarily used to test and measure activity levels in the recovered population, but it cannot effectively measure physical performance indicators in young people with good motor function (Treacy and Hassett, 2018).

### BBS

The BBS consists of 14 items, each of which is scored from 0 (unable to perform) to 4 (normal execution) on a scale of 0–56, involving functional balance control, including transfer, steering, and walking, with a higher score indicating better balance (Berg et al., 1992). BBS is a reliable, effective, and widely used tool. It does not require people to be able to walk or stand independently. It has often been used to evaluate the body balance ability of people with various physical conditions and disabilities, such as poststroke patients (Moon et al., 2017; Ursin et al., 2019), and the BBS can also predict the loss of important activities in daily life (Wennie Huang et al., 2010). However, BBS also has limitations. The BBS cannot measure gait quality and walking speed and is not suitable for screening patients under 75 years old (Downs, 2015). The evidence supporting the use of BBS to predict falls is insufficient, and it should not be used solely to determine the risk of falls in the elderly (Lima et al., 2018).

### TUG

The TUG test is a reliable and effective rapid measurement method for evaluating athletic ability and quantifying athletic performance. This test records the time it takes subjects to sit back down after completing a 3-m walk and returning to the armchair, involving the movement speed and trunk balance (Wu et al., 2019). The TUG test has been shown to have a good correlation with the BBS (Podsiadlo and Richardson, 1991), but it is more convenient and commonly used to assess senior functional activities after stroke (Chan et al., 2017). TUG can assess realistic mobility, including potential falls (Bischoff et al., 2003). However, the TUG test has some limitations. It does not address the problem of decreased motor performance when performing cognitive and motor tasks at the same time (Plummer et al., 2013). The latest meta-analysis suggests that TUG should not be used alone to predict fall risk (Park, 2018).

### TMT

TMT consists of nine balance tests (0–16 points) and eight gait tests (0–12 points), with a higher total score indicating better balance and motor function. TMT is a reliable and effective method for assessing mobility, balance and gait, as well as predicting the risk of falling (Kopke and Meyer, 2006; Panzer et al., 2011). TMT has been widely used in studies of older people, as well as in other diseases, such as Parkinson’s disease and stroke (Alvarez et al., 2020; Perez-de la Cruz, 2020). However, recent studies have shown that TMT alone does not predict fall risk in older adults (Omana et al., 2021).

## Diagnosis and Treatment

Gait and balance instability are important factors that cause falls in older adults (Wei et al., 2017; Bower et al., 2019), so screening out patients with CSVD with impaired gait and balance function and intervening with pharmacological and nonpharmacological interventions is important to improve the prognosis of CSVD.

### Diagnosis and Identification

Walking speed is a very meaningful parameter for assessing the health of older adults, and if a person’s spontaneous walking speed is below 0.6 m/s, it is considered problematic (Panzer et al., 2011; Studenski et al., 2011). A total TMT score <19, ^3^BBS scores < 40 (Muir et al., 2008), and TUG ≥ 13.5 s (Clemson et al., 2015) indicate a higher risk of falling. A simple balance test, such as recording the time spent standing on one leg or in tandem, can also be used to identify CSVD patients at risk of falling early and assess their balance function (Jahn et al., 2015). Gait degradation under dual-task requirements is typical of gait disorders with cortical and subcortical involvement. Setting up dual tasks can catch defects at an early stage and suggest preventive measures such as increasing physical activity by increasing daily walking distance (Montero-Odasso et al., 2012). Subjective visual vertical tests are helpful in patients with unstable gait and a tendency to fall and are simple to perform (“bucket test”; Jahn et al., 2015).

### Rehabilitation Therapy

In patients with impaired gait and balance, early rehabilitation is necessary. Traditional gait training methods include one-legged weight-bearing, the center of gravity shifting, stepping and hip stretching training, using the affected limb to go up and down stairs, walking laterally, and walking in place (Brock et al., 2011). Balance training included standing on one leg, walking in a straight line, throwing and catching the ball, standing in front of the mirror and being pushed in different directions by the therapist, and moving the body’s center of gravity forward, backward, sideways, and obliquely when the eyes were open and closed (Chung et al., 2014). The pro-kin system is a new visual feedback balance training method. The combination of pro-Kin system and conventional rehabilitation therapy can improve the gait and balance function of CSVD patients without obvious cognitive impairment better than traditional rehabilitation training (You et al., 2017; Zhao et al., 2019). In addition to physical exercises such as walking, people can also try exercise cognitive training such as tai chi and Dalcroze music pedagogy to improve the executive function of the elderly (Jahn et al., 2015).

### Treatment of CSVD

Blood pressure is the most important controllable risk factor for CSVD. Intensive antihypertensive therapy can reduce the progression of WMH but the effect on brain atrophy is unclear (van Middelaar et al., 2018; Su et al., 2021). Intensive blood pressure lowering reduced the recurrence of stroke in patients with LI through a multicenter clinical trial called SPS3 (SPS3 Study Group et al., 2013). A more aggressive antihypertensive regimen may be justified, but too low a blood pressure carries the risk of cognitive impairment (Webb et al., 2010). Blood pressure variability is associated with WMH, CMB, EPVS, and total CSVD burden and may also lead to a poor prognosis. Therefore, reducing ambulatory blood pressure variability is important for the control of CSVD (Chen et al., 2019; Zhang et al., 2019a).

Patients who received intravenous r-tPA had a better neurological prognosis than those who received a placebo in LI (Mok and Kim, 2015). The presence of CMB and WMH increases the risk of symptomatic cerebral hemorrhage and poor prognosis, but patients still have a net benefit (Charidimou et al., 2016). The benefits and risks of intravenous thrombolysis in patients with CSVD need to be assessed individually to minimize the occurrence of cerebral hemorrhage and poor prognosis after thrombolysis in ischemic stroke.

For antiplatelet therapy, refer to the 2018 American Heart Association/American Stroke Association published guidelines for the early management of patients with acute ischemic stroke (Powers et al., 2019). Given the bidirectional nature of CSVD with both ischemia and bleeding risk, an assessment of treatment benefits and bleeding risk should be performed. Antiplatelet agents should be used with caution in patients with severe WMH and a large number of CMB. In patients with severe WMH and multiple CMB, cilostazol may be a safer option compared to aspirin and clopidogrel (Kim et al., 2020).

Statins have been shown to lower lipids, reduce oxidative stress and protect the vascular endothelium (Sander et al., 2005; Erdos et al., 2006), which may reduce the risk of stroke recurrence including ischemic stroke due to CSVD (Amarenco et al., 2009). Studies have shown that the application of statins can reduce the progression of WMH and the decline in cognitive function (Xiong et al., 2014). However, in patients with severe CSVD, high doses of statin are associated with an increased risk of a cerebral hemorrhage (Amarenco et al., 2009).

Daily B vitamins can lower homocysteine and thus may reduce WMH volume progression in patients with severe CSVD (Cavalieri et al., 2012). In addition, vitamin E tocotrienols slow the progression of WMH in subjects with brain white matter injury (Gopalan et al., 2014). Prevention and treatment strategies for CMB should target cerebral microvascular endothelium and function, blood-brain barrier, and neuroinflammation, such as endothelin antagonists, peroxisome proliferator-activated receptor-agonists, and neurotrophic factors (Nagasawa et al., 2014).

### Therapy for Cognition and Depression

CSVD-associated cognitive dysfunction is an important subtype of vascular cognitive dysfunction and is one of the common causes of vascular dementia. There is some degree of overlap in the neuropathology of AD and vascular dementia, and cholinergic insufficiency may lead to vascular cognitive impairment (Roman and Kalaria, 2006). Various drugs, including donepezil and galantamine, have shown modest cognitive benefits in patients with vascular dementia. However, the benefits of other drugs, such as rivastigmine, memantine, nimodipine, piracetam, and herbal remedies, remain unclear (Farooq et al., 2017). Large randomized controlled trials have shown benefits in improving cognitive function in patients with mild to moderate vascular cognitive impairment at 24 weeks, and patients tolerate donepezil well (Goldsmith and Scott, 2003; Malouf and Birks, 2004). Nevertheless, in patients with CADASIL alone, donepezil did not significantly improve overall cognitive function (Dichgans et al., 2008). After 6 months of galantamine use, patients with mild to moderate cognitive impairment with AD and vascular dementia had significant improvements in cognitive function, but the side effects were significant (Erkinjuntti et al., 2002; Auchus et al., 2007). There are also small sample studies that have found that antihypertensive drugs, statins, and nimodipine drugs may have some effect on CSVD-related cognitive impairment, but more large randomized controlled trials are needed to confirm this (Zhang et al., 2019b; Zhang J. et al., 2019). Patients with vascular depression responded poorly to common antidepressants, and the response rate of combination therapy with citalopram and methylphenidate was higher than that of the drug alone. This treatment also leads to improved function (Lavretsky et al., 2015; Smith et al., 2021). In elderly patients with depression and cognitive impairment, the combined use of antidepressants and donepezil had a temporary positive effect on cognitive function but increases the risk of recurrence (Reynolds et al., 2011).

## Discussion

In conclusion, exploring the correlation between CSVD and gait and balance disorders is of great clinical significance for the diagnosis and treatment of gait and balance disorders and the prevention of falls. CSVD is a disease in which multiple pathological changes can coexist, and its influence on gait and balance function is not caused by a single factor but may be the result of the joint action of multiple factors. In addition, imaging features of different CSVD and injury of different parts may lead to different characteristics and mechanisms of gait and balance injury but there may be overlap between them. And different small vascular diseases may also affect each other. In general, CSVD can affect gait and balance by affecting cognitive function, disrupting gait structures associated with balance control, influencing depressive states, and interactions. Rehabilitation and medication may play a role in gait and balance disorders.

Currently, most studies on CSVD and gait and balance disorders are cross-sectional studies or retrospective studies, and more prospective studies with large samples are needed in the future to further clarify the correlation between the two. In terms of the crowd, future studies should stratify patients with different causes and severity of the disease, such as the mild stroke population (Loos et al., 2018). The application of advanced imaging technology, such as VBM technology and DTI technology, can be a more accurate and comprehensive evaluation of the imaging appearances of CSVD, to clarify the role of CSVD in different brain diseases. As for the evaluation methods of gait and balance function, new measurement methods such as electronic sensors should be applied in gait and balance evaluation in the future, so as to obtain a more real and comprehensive evaluation and reduce the results bias caused by different test methods. Regarding the treatment of CSVD-related gait and balance disorders, future large-scale randomized controlled trials are needed to determine the direct effects of medication or rehabilitation on gait and balance function. The mechanisms of CSVD affecting gait and balance need to be further clarified, so as to provide more perspectives for the prevention and treatment of gait and balance disorders.

## Author Contributions

RZ planned the study. CS, XY, and SW analyzed the data and edited the manuscript. CS wrote the manuscript. All authors contributed to the article and approved the submitted version.

## Conflict of Interest

The authors declare that the research was conducted in the absence of any commercial or financial relationships that could be construed as a potential conflict of interest.

## Publisher’s Note

All claims expressed in this article are solely those of the authors and do not necessarily represent those of their affiliated organizations, or those of the publisher, the editors and the reviewers. Any product that may be evaluated in this article, or claim that may be made by its manufacturer, is not guaranteed or endorsed by the publisher.
